# Integrating Multi-Omics for Uncovering the Architecture of Cross-Talking Pathways in Breast Cancer

**DOI:** 10.1371/journal.pone.0104282

**Published:** 2014-08-19

**Authors:** Li Wang, Yun Xiao, Yanyan Ping, Jing Li, Hongying Zhao, Feng Li, Jing Hu, Hongyi Zhang, Yulan Deng, Jiawei Tian, Xia Li

**Affiliations:** 1 College of Bioinformatics Science and Technology, Harbin Medical University, Harbin, China; 2 Department of Ultrasonic medicine, The 2nd Affiliated Hospital of Harbin Medical University, Harbin, China; 3 Department of Ultrasonic medicine, The 1st Affiliated Hospital of Heilongjiang University of Chinese Medicine, Harbin, China; Univesity of Texas Southwestern Medical Center at Dallas, United States of America

## Abstract

Cross-talk among abnormal pathways widely occurs in human cancer and generally leads to insensitivity to cancer treatment. Moreover, alterations in the abnormal pathways are not limited to single molecular level. Therefore, we proposed a strategy that integrates a large number of biological sources at multiple levels for systematic identification of cross-talk among risk pathways in cancer by random walk on protein interaction network. We applied the method to multi-Omics breast cancer data from The Cancer Genome Atlas (TCGA), including somatic mutation, DNA copy number, DNA methylation and gene expression profiles. We identified close cross-talk among many known cancer-related pathways with complex change patterns. Furthermore, we identified key genes (linkers) bridging these cross-talks and showed that these genes carried out consistent biological functions with the linked cross-talking pathways. Through identification of leader genes in each pathway, the architecture of cross-talking pathways was built. Notably, we observed that linkers cooperated with leaders to form the fundamentation of cross-talk of pathways which play core roles in deterioration of breast cancer. As an example, we observed that KRAS showed a direct connection to numerous cancer-related pathways, such as MAPK signaling pathway, suggesting that it may be a central communication hub. In summary, we offer an effective way to characterize complex cross-talk among disease pathways, which can be applied to other diseases and provide useful information for the treatment of cancer.

## Introduction

During the last decade, researchers have witnessed the complexity and redundancy of molecular mechanisms in mammalian cells [1], [2]. Such complexity and redundancy are mostly attributed to the cross-talk among various biological pathways [3]. Cross-talking pathways can communicate with each other in diverse regulatory ways, such as feedback circuits, to produce complex physiological reactions to maintain normal operation of stable biological systems [4], [5]. Importantly, the development of cancer is also dependent on complex cross-talk among abnormal biological pathways, which extremely increase the difficulty of cancer treatment, since their complexity and redundancy. The inhibition of only one or a few target genes cannot restore the abnormal cross-talk of pathways and thus cannot achieve the desired treatment outcomes [4]. It is therefore important to systematically understand the complex pathogenesis underlying human cancers through considering cross-talk of risk pathways. Recent studies have begun to reveal the cross-talk between various biological pathways; however, these represent only the tip of the iceberg.

Complicatedly, cancerogenesis is generally related to molecular changes at multiple levels, including genomics [6], DNA methylomics [7], and transcriptomics [8]. Somatic mutation and copy number aberrations (CNAs), as hallmarks of cancer, play an important role in the development, progression and prognosis of cancer by deregulating gene expression and increasing chromosomal instability [9], [10]. DNA methylation provides new insight into the pathogenesis of cancer and is considered as an emerging biomarker for cancer detection, diagnosis and treatment [11], [12]. Aberrant DNA methylation in promoters of oncogenes or tumor suppressor genes has been observed in multiple cancer types [13], [14]. In addition, extensive expression changes in cancer have been widely characterized. It is thus insufficient to dissect the pathogenesis of cancer only from single molecular levels.

The accumulating genome-wide data at multiple molecular levels have been generated using a variety of high-throughput technologies, such as SNP array, DNA methylation and expression microarrays, especially from the same samples. Integration of multi-omics data has been applied to a variety of human diseases [15]–[18], such as breast cancer [19]–[21], leukemia [22], and glioblastoma [23], for revealing their potential mechanisms. Setty et al. proposed a regularized regression to identify important regulators in the expression of glioblastoma by integrating multidimensional data [24]. Multiple concerted disruption (MCD) analysis was used to calculate the explaining variance of different factors in the differential gene expression and identify cancer-associated genes and pathways [21]. Therefore, the molecular dysfunctions of cancer at multiple levels should be combined to systematically identify the cross-talk among the risk pathways in cancer.

**Figure 1:**
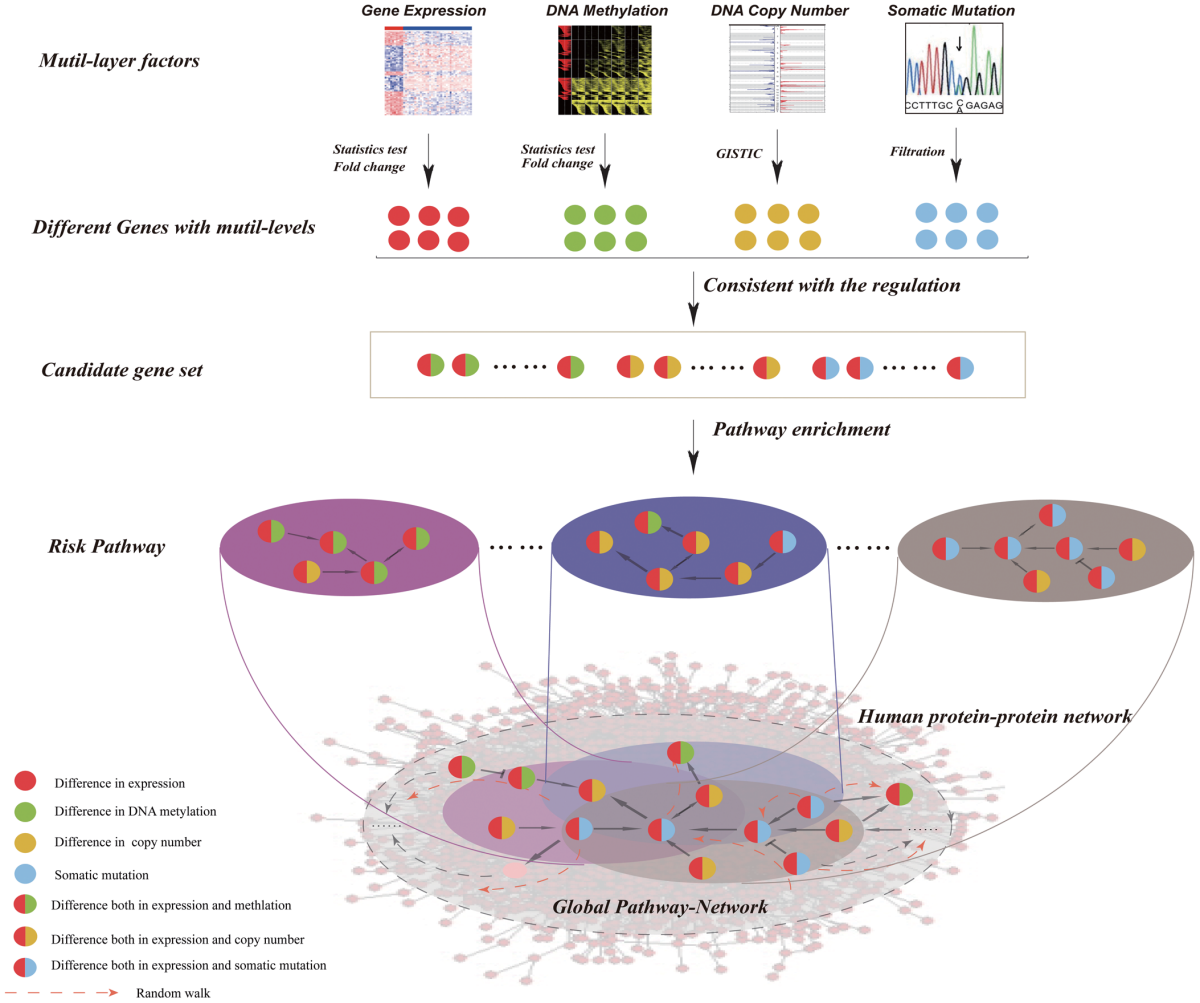
The workflow of identifying cross-talk among risk pathways. Step 1: Integration of genomic, DNA methylomic, and transcriptomic data for identification of candidate genes; Step 2: Identification of risk pathways by using candidate genes based on pathway enrichment analysis; Step 3: Identification of cross-talk among risk pathways by mapping candidate genes onto the human protein interaction network based on the random walk with restart method.

In this study, we present a computational strategy for constructing a pathway cross-talk network based on random walk of candidate genes in a protein–protein interaction network by integrating multidimensional genome data (Figure 1). By application of our method to multi-omics data of breast cancer from TCGA, we identified risk pathways by considering different combinations of molecular changes at different levels, and then built the pathway cross-talk network associated with breast cancer. Our results showed numerous cross-talks between known cancer-related pathways and identified linkers by network topological analyses. Then we constructed the architecture of cross-talking pathways that was composed of linkers and leaders, and revealed how these linkers and leaders mediate the cross-talk among different pathways.

## Materials and Methods

### Multi-dimensional breast cancer genomic data

The multi-dimensional breast cancer associated datasets containing 304 human breast cancer samples and 18 non-tumor samples with mRNA expression data, DNA methylation, DNA copy number, and somatic mutation, which were collected from the public database TCGA (available at https://tcga-data.nci.nih.gov/docs/publications/brca_2012/). The level 3 data of gene expression profile, detected by Agilent 244K, contained 17815 genes. The level 3 data of DNA methylation data were obtained by using the Infinium HumanMethylation27 BeadChip kit, which detected the methylation levels of 27,578 CpG sites in the promoters of 14,475 genes. The methylation level of each CpG site was calculated as the ratio of the signal of methylated probes relative to the sum of methylated and unmethylated probes, which ranged continuously from 0 (unmethylated) to 1 (fully methylated). For a CNA array data (SNP 6.0), the segmentations were identified using the circular binary segmentation method based copy numbers estimated by calculation of normalized log2-tranformed ratios (level 3) [25]. We also extracted a somatic mutation data (level 2) from TCGA. Through removing silent mutations, a total of 8543 somatic mutation genes were obtained. The common genes detected by gene expression, DNA methylation, copy number and somatic mutation profiles were used for subsequent analysis.

The protein–protein interaction network was downloaded from the Human Protein Reference Database (HPRD), which includes 9219 genes and 36900 interactions among those genes. We retrieved the largest connected component of the protein-protein interaction network for subsequent analysis.

### Identification of candidate genes with alterations at multiple levels

Considering the asymmetric cancer and control samples, we performed 1000 random sampling to identify significantly differentially expressed genes and significantly differentially methylated genes. For each sampling, we identified a set of differentially expressed genes by comparing the expression profiles of 18 randomly selected cancer samples from 304 cancer samples with 18 normal samples using t-test (Benjamini-Hochberg adjusted FDR<0.001, fold change>2). Next, for each gene, we computed the frequency of significant difference in 1000 random sampling. The gene with the frequency greater than 10% was regarded as a significantly differentially expressed gene in breast cancer. As for DNA copy number data, we applied the GISTIC (version 2) to identify genes with genomic amplification and deletion in breast cancer with default parameters.

### Identification of risk pathways

By combination of dysregulation at different molecular levels, we identified three groups of candidate genes: (i) genes with differential expression and differential methylation (i.e. overexpression and loss of DNA methylation, underexpression and gain of DNA methylation); (ii) genes with differential expression and copy number alteration (i.e. overexpression and amplification, underexpression and deletion); and (iii) genes with differential expression and somatic mutation. Finally, we used these three gene sets to identify subpathways which were key regions impacted by candidate genes within all human pathways from KEGG, with Benjamini-Hochberg adjusted FDR<0.05, using an R package iSubPathwayMiner (version 3.0, available at http://cran.r-project.org/web/packages/iSubpathwayMiner/) [26]. Briefly, the subpathway recognition procedure consists of three steps. (i) Candidate genes were mapped to all pathways; (ii) Subpathways within pathways were located according to candidate genes. In this step, we firstly computed the shortest path between candidate genes for each pathway, and merge candidate genes and other genes based on their shortest path *n*. Subsequently, we extracted the corresponding subgraph according to gene sets and deleted the subgraphs in which the number of genes is less than *s*. (iii) The statistical significance of subpathways was evaluated. These significantly enriched subpathways were termed as “risk pathways”.

### Construction of pathway cross-talk network based on protein interaction network by random walk

To detect the cross-talk among risk pathways, we used the human protein interaction network, which provides a possible way to reveal the functional information flow among biological pathways. To globally consider the topological structure of the protein interaction network, we used random walk with restart (RWR) [27], in which the information flow has a certain probability to get back to the information source at every walk step, to measure the strength of cross-talk between any pairs of risk pathways.

For each pair of risk pathways, we considered the risk genes in one pathway as the information source (i.e., source nodes) and those in the other pathway as the information target (i.e., target nodes). The information flow starting from the set of source nodes iteratively and randomly walk through the protein interaction network by transmitting to their neighbors with a probability that is proportional to their topological features. At each step, the information flow can return to the source nodes with some probability. The mean steady state probability that the source nodes will finally stay at the target nodes is calculated to reflect the strength of cross-talk between the two paired pathways. Random walk with restart is a recursive procedure, which can be represented as follows:
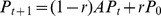
where *P_0_* is the initial probability of functional information for all nodes in the protein interaction network; *P_t_* and *P_t+1_* are the probability of functional information for all nodes at the *t*-th and (*t*+1)-th steps, respectively; *A* represents the column-normalized adjacency matrix corresponding to the protein interaction network, in which the sum of each column is 1; and *r* is the restart probability, which indicates the chances of information flow back to the seed nodes.

To address the significance of the strength of cross-talk from the source nodes to the target nodes, 1000 random networks were generated from the protein interaction network by keeping the degree of nodes unchanged. We recalculated the strength of cross-talk between the pathways in the 1000 random networks using random walk and obtained 1000 scores. The *p values* that consider the number of random values that were greater than the observed statistics were then computed. Statistically significant cross-talk was established at p<0.05, which was corrected for multiple hypothesis testing by applying a Benjamini-Hochberg correction using the p.adjust function in the R stats package. Finally, we collected the significant cross-talks between pathway pairs and then to assemble them into an undirected pathway cross-talk network by reasonable optimization.

### Linkers and leaders in cross-talking pathways

**Figure 2:**
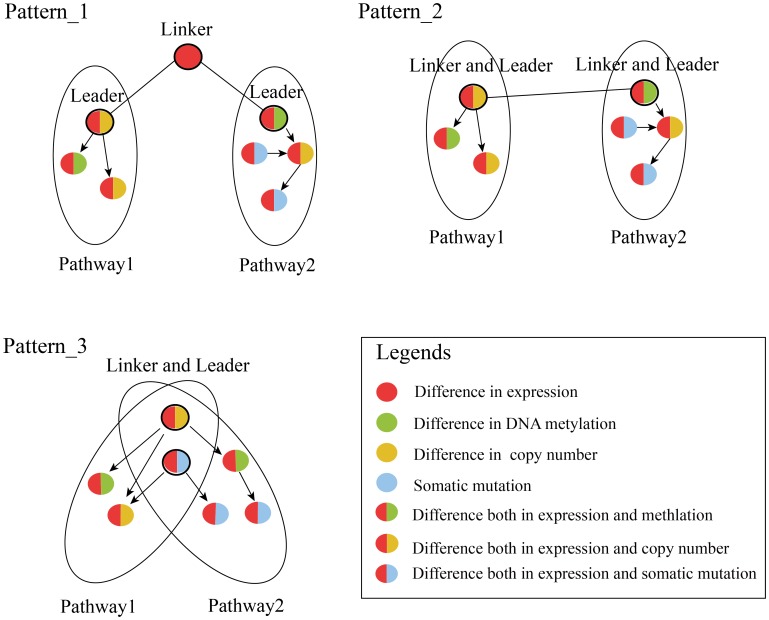
Linkers and leaders in cross-talking pathways. For cross-talking pathway pairs, the linker and leader genes show three situations. The black circles with different color represent the linker and/or leader genes with the alteration in different levels.

Given two cross-talking pathways *A* and *B*, candidate genes G_A_ and *G_B_* are respectively involved in these two pathways. We mapped these dysfunctional genes *G_A_* and *G_B_* into protein interaction networks and then identified the linkers and leaders. The identification of linkers and leaders were based on cross-talk between risk pathways. Linkers are defined as genes that show significant alteration for at least one molecular level and can mediate two cross-talking risk pathways. Leaders are defined as candidate genes in risk pathways that can directly connect the linkers. For each cross-talking pathway pair, the linkers and leaders showed in three situations: (i) Genes that show alteration at single molecular level and are not members of risk pathways directly connect candidate genes in the risk pathways, which are termed linker genes. These connected candidate genes are termed leader genes; (ii) Candidate genes in the cross-talking risk pathways directly connecting each other are considered as both linker and leader genes; (iii) In the case of cross-talking pathway pairs with an overlap of candidate genes, these overlapping candidate genes are recognized as both linker and leader genes (Figure 2).

## Results

### Identifying abnormal genes in breast cancer at multi-levels

**Figure 3:**
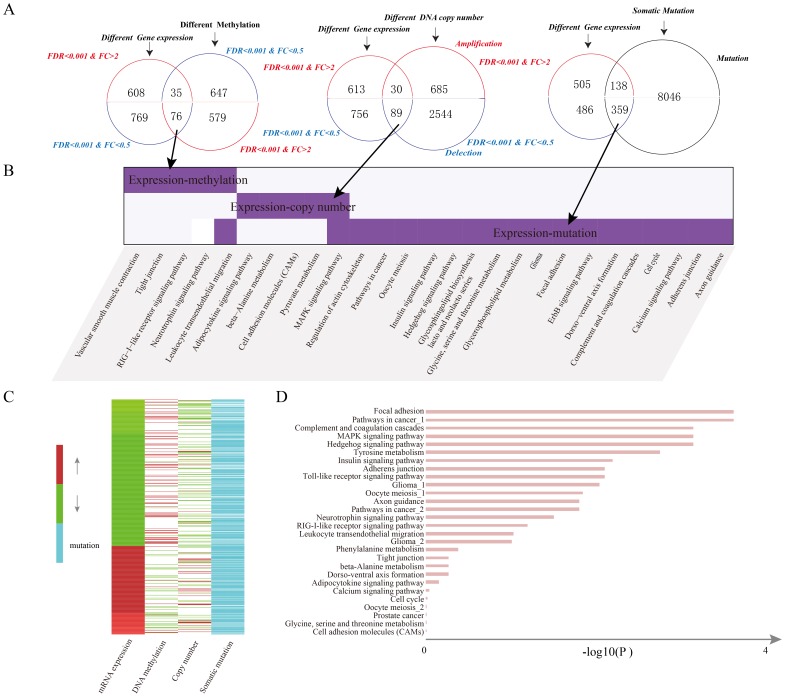
Integrative information of candidate genes with alterations at multiple levels and risk pathways. (**A**) Venn diagram showing the dysregulated groups between mRNA expression and another molecular level such as DNA methylation,DNA copy number and somatic mutation. (**B**) Comparisons among pathways enriched by potential candidate genes derived from different dysregulated groups. (**C**) The alteration patterns of candidate genes in at least two levels. (**D**) The significantly enriched risk pathways by candidate genes (*FDR*<0.05).

We obtained gene expression, DNA methylation, DNA copy number, and somatic mutation data on 304 human breast cancer samples and 18 normal samples, from TCGA. Comparing the tumor samples with normal samples, we identified 1488 differentially expressed genes and 1337 differentially methylated genes (Table S1 and S2). Also, 3348 genes showing significantly recurrent copy number alterations were identified by GISTICv2.0. Consistent with previous findings [21], only a few genes showed abnormalities at the multi-levels. We compared the dysregulated genes and found complex patterns of molecular abnormalities at different levels. We thus categorized these genes into three dysregulated groups (Figure 3A): (i) 35 genes that show overexpression and hypomethylation; and 76 genes that show underexpression and hypermethylation; (ii) 30 genes that show overexpression and copy number amplification; and 89 genes that show underexpression and copy number loss; (iii) 497 genes with somatic mutation and differential expression (138 overexpression and 359 underexpression). The complex patterns suggested that there may exist a mass of key genes which change at different molecular levels together to form the molecular mechanisms underlying cancer [28].

### Establishing candidate genes by combining information from multiple levels

We performed pathway enrichment analyses on those genes in the three dysregulated groups respectively, and identified significantly enriched pathways at *p* value<0.01 (Figure 3B). We found that genes in each dysregulated groups were involved in many important cancer-related pathways, such as the “MAPK signaling pathway”, “RIG–I–like receptor signaling pathway” and “pathways in cancer”. It is interesting to note that significantly enriched pathways in different groups showed low consistency. This suggested that genes dysregulated at single molecular levels could only capture parts of the whole cancer-related molecular network and that integration of genes with changes at different levels is necessary to understand the complex mechanisms of cancer [18], [29], [30]. Therefore, we selected all of the 730 genes in the three dysregulated groups as the candidate genes.

Of these candidate genes, we found HOXA2, HOXA4, HOXA5, HOXA7, and HOXA9 showing underexpression with hypermethylation. Aberrations in members of HOX genes have been reported in abnormal development and malignancy, indicating that altered expression have tumorigenic potential and thus, are often defined as ‘cancer genes’ [31], which consistent with our result. CHL1, a potential tumor suppressor gene, was found to be downregulated in expression and to show hypermethylation, in line with a recent report of its high association with early preinvasive growth of breast cancer through facilitating tumor growth [32]. Moreover, we revealed the copy number amplification and mRNA overexpression of ARF1, suppression of which could result in the inhibition of breast cancer cell migration and proliferation [33] (Figure 3C).

### Constructing the network of cross-talking pathways in breast cancer

We analyzed 730 candidate genes using iSubpathwayMiner 3.0 with the parameters *s* = 5 and *n* = 5 and then identified the risk subpathways in breast cancer at Benjamini-Hochberg adjusted FDR<0.05 (Table S3). We set *s* = 5 because previous studies reported that subpathways (s≥5) were associated with disease and considered to represent a pathway. Our previous study revealed that 85% of disease genes was with shortest path <5 to its nearest disease gene suggesting a value of *n* = 5 seems to represent the closeness of candidate genes [26].

In total, twenty eight biological pathways were identified, such as “Focal adhesion”, “Pathways in cancer”, “Complement and coagulation cascades”, “MAPK signaling pathway”, and “Hedgehog signaling pathway” (Figure 3D). We found significantly enriched many important pathways such as “Pathways in cancer”, “Cell adhesion molecules (CAMs)”, and “Toll-like receptor signaling pathway” which play a core role in the activation of immune responses emerged as attractive targets for therapeutics in cancer [34], [35]. These pathway showed complex abnormal patterns at different molecular levels, including somatic mutation, DNA methylation and copy number alterations. Notably, “Toll-like receptor signaling pathway” was identified by combination of these three sets of candidate genes indicating the importance of integrations of multi-level information.

**Figure 4:**
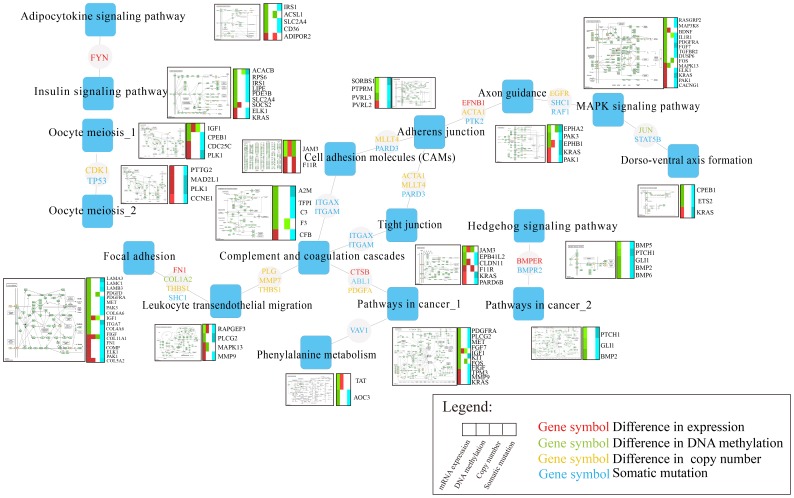
The network of cross-talking pathways. The cross-talking pathways with the alteration patterns of candidate genes in each risk pathway and linkers mediating cross-talk of pathway pairs are shown. Each square node represents the risk pathway and the circle made of linkers. The different colors of linkers represent the change in different molecular level.

To identify the cross-talk between two risk pathways, we used the protein interaction network to evaluate whether the candidate genes in the two pathways have close connections within the network. We attempted to construct a directed pathway cross-talk network among 28 risk pathways. Using RWR, the cross-talk scores of 378 pairs of pathways from among the 28 pathways were computed. Based on those scores from 1000 randomly permutated networks,15 significant cross-talk pairs of pathways were determined at Benjamini-Hochberg adjusted FDR<0.05 (Table S4). The 15 cross-talk pairs involved 17 risk pathways. To further investigate genes mediating the cross-talk among the risk pathways, we mapped the candidate genes from the cross-talk pathways onto the protein interaction network and selected the common direct neighbors of candidate genes in each pathway pair in the PPI network (Figure 4).

### Linkers mediating cross-talking pathways

For these 15 cross-talking pathway pairs, we identified 73 linkers and 46 leaders. The number of linkers in each cross-talking pair is from 1 to 18, with an average of 4. And the number of leaders in each pair is from 2 to 12, with an average of 3 (Table S5). For example, a linker gene JUN connecting two leader genes FOS and ETS2 (FOS/JUN/ETS2) mediates cross-talk between “MAPK signaling pathway” and “Dorso-ventral axis formation” (Figure S1). It has been verified that JUN can interact with FOS as a complex which subsequently interacts with ETS2 to form the stable heterotrimeric FOS/JUN/ETS2. The FOS/JUN/ETS2 plays important roles in tumor invasion and metastasis [36].

The linkers act as key genes and play important roles in mediating cross-talk between risk subpathways. We performed survival analysis for each differentially expressed linker and evaluated the significance of correlations between these linkers and overall survival using univariate cox regression analyses. We found that two linker genes COL4A6 (p = 0.01) and PLK1 (p = 0.07) whose expressions are weakly associated with the survival of breast cancer patients. Then, we divided the patients into two different groups based on the median expression of COL4A6 and PLK1, respectively. Kaplan-Meier curves of different groups suggest the low expression of COL4A6 and high expression of PLK1 are both associated with poor survival (p<0.1 for both COL4A6 and PLK1, log-rank test) (Figure S2). COL4A6, a major component of the basement membrane, plays an important role in confinement of the tumor microenvironment [37]. The down-regulated expression of COL4A6 has been certified in breast cancer [38]. PLK1 acts as a proto-oncogene through driving cell cycle progression. Lee et al. [39] reported that PLK1 might alter BRCA2 function by phosphorylating BRCA2 in breast cancer, which could subsequently lead to genetic instability. It may provide a possible functional explanation that overexpression of PLK1 are associated with poor survival in breast cancer, suggesting a role for PLK1 as a potential prognostic marker.

Further, for each pair of cross-talking pathways, we identified the biological processes significantly enriched by the candidate genes in each pathway and linkers respectively (Figure S3). We observed the same or similar biological processes between each pathway pairs and linkers. For example, the linkers (MLLT4 and ACTA1) mediated the cross-talk of “Tight junction” with “Adherens junction” through regulating important cell functions in cancer, such as “cell motility/migration [40]”, “cell adhesion [41]” and “cell junction assembly [42]”. The function consistence between cross-talking pathways and linkers supported the connection of the linkers with the cross-talking pathways, which provide implications that the close cross-talks of risk pathways are crucial to the cancer progression.

### Discovering the architecture of cross-talking pathways

**Figure 5:**
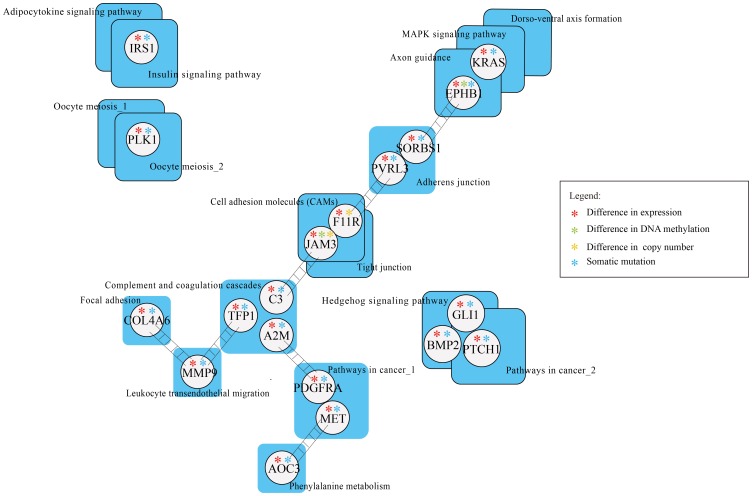
The architecture of cross-talking pathways. The blue squares represent risk pathways in the network of cross-talking pathways and the lightly grey circles with different color asterisks represent the leaders with the alteration in different levels.

Based on function connection of linkers and their mediating cross-talking pathways, we identified the architecture of cross-talking pathways which presented a blueprint of the potential pathogenesis of breast cancer (Figure 5).

**Figure 6:**
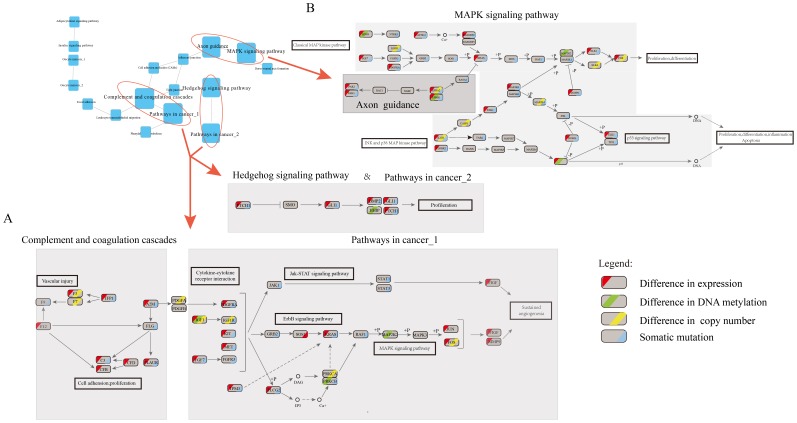
Integrative pathways from different cross-talking pathway pairs. (**A**) “Complement and coagulation cascades” and “Pathway in cancer_1”, “Hedgehog signaling pathway” and “Pathway in cancer_2”; (**B**) “Axon guidance” and “MAPK signaling pathway”. Each ellipse represents a gene in KEGG pathway or ppi network. For a given gene, the ellipse is divided into four parts (from left to right) corresponding to different molecular level with abnormal states (red) in mRNA expression, (green) DNA methylation, (yellow) copy number and blue (somatic mutation), respectively.

As expected, we found that significantly enriched subpathways from the same pathway of close cross-talking, such as subpathways Meosis I and Meosis II in the pathway Oocyte meiosis shared a common gene PLK1, which showed overexpression and somatic mutation. Consistently, overexpression of PLK1 and knockdown of its linker TP53 can enhance the stability of CEP55 which could regulates breast cancer progression and metastasis [36]. Notably, we observed that subpathways from the same pathway contributed to specific biological processes by cross-talking with different pathways. The subpathways “Pathway in cancer_1” and “Pathway in cancer_2” from the same pathway had no connection, but linked to two distinct risk pathways (including “Complement and coagulation cascades and Hedgehog signaling pathway), respectively. The pathway “Complement and coagulation cascades” cross-talk with “Pathway in cancer_1” through the interaction of leaders (A2M and PDGFRA) with linkers (PDGFA), and ultimately result in sustained angiogenesis (Figure 6A). Both A2M and PDGFRA with underexpression and somatic mutation can lead to the cancer development [43], [44]. Also, PDGFA was more frequently expressed in breast tumors [45]. Analogously, “Hedgehog signaling pathway” cross-talk with “Pathway in cancer_2” to promote proliferation by shared dysfunctional linkers and leaders such as PTCH1, BMP2 and GLI1 all of which show under-expression and mutation. Meanwhile, another two leaders (TFP1,C3) in “Complement and coagulation cascades” showed the highest degree linked with different pathways (including “Leukocyte transendothelial migration”, “Cell adhesion molecules (CAM)s”, and “Tight junction”). Consistently, previous studies have validated that “Complement and coagulation cascades” interacts with several cancer associated pathways and then affect pathway function. These findings suggested that the leader in “Complement and coagulation cascades” pathway may be a communication hub into other pathways, which play an important role in breast cancer [46], [47].Some dysfunctional genes (including KRAS, F11R, JAM3 and PDGFRA) with different molecular alterations have participated in multiple pathways (such as “MAPK signaling pathway”, “Cell adhesion molecules (CAMs) and Pathways in cancer”), which suggests that these leaders serve as exchange centers for the cross-talk among the pathways. In particular, KRAS, a known oncogene [48], was found to be involved in 6 of the 17 risk pathways. Our result showed that the dysfunction of KRAS through mutation mediated the cross-talk of “Axon guidance” with “MAPK signaling pathway” (Figure 6B). We further found that dysfunction of KRAS may result in up-regulation of its downstream target ELK1 to promote proliferation and differentiation, consistent with previous findings [49]. Notably, JAM3, which participates in cell-cell adhesion, and may also be utilized by cancer cells to promote tumour progression or survival [38], [50], showed not only hypermethylation but also copy number loss accompanied by under-expression.

## Discussion

The accumulation of high-throughput, multidimensional, genome-wide data provides an opportunity to characterize the complex molecular mechanisms underlying cancers. In our study, we proposed a systematic strategy that integrates genomic, methylomic, and transcriptomic data to identify the cross-talk among risk pathways based on alterations of genes at different molecular levels through random walk in the protein–protein interaction network. We applied our method to a large scale of breast cancer samples, referring to multi-Omics data including gene expression profiles, DNA methylation, DNA copy number and somatic mutation, for constructing the network of cross-talking pathways in breast cancer. Our approach successfully identified many known cross-talks between cancer-related pathways. Furthermore, using the network of cross-talking pathways, we determined many linkers bridging these cross-talks and leaders in the architecture of cross-talking pathways. For example, KRAS showed a close association with many risk pathways and appeared to function as an inter-hub among pathways, mediating cooperation between different risk pathways.

Our method focused on the cross-talk among multiple risk pathways rather than among only a few genes or pathways at single molecular levels. Such multilevel-based integration strategies can provide more information about the pathogenesis mechanism of cancer and thus can be helpful in distinguishing driver genes from passengers. A number of studies have shown the power of integration of information from multiple molecular levels [21], [24], [51]. Our results strongly show that different change patterns occur in many known cancer-related pathways, which in turn form tight cross-talks among these pathways, further supporting the necessity of integrative analysis.

Our method not only integrated multidimensional genomic data but also considered the topological structure of candidate genes in the protein interaction network. The protein interaction network reflects the functional association among genes. Genes located close to each other tend to show similar functions, thus providing a reliable resource to reveal the cross-talk among pathways. To consider the functional similarity of genes from the topological perspective, we used random walk with restart to quantify the cross-talk between pairs of pathways. Previous studies have shown that random walk approaches individually outperform other methods, such as neighborhood approaches, in the prediction of gene–disease associations based on the protein interaction network [52], [53]. This might be attributed to the fact that the random walk method offers the advantage of combining candidate genes with functional network topology by tracing the flow of functional information in the protein interaction network.

In conclusion, the identification of cross-talk between risk pathways using multidimensional genomic data is useful in characterizing the potential mechanism of human cancer. With the increasing availability of multidimensional genomic data, our method can integrate information from more levels and detect novel and important leader genes and linkers that mediate the cross-talk between risk pathways, which will provide useful information on target treatment of cancer.

## Supporting Information

Figure S1 **The function network of cross-talking pathways.** Based on the structure of cross-talking pathways, the significantly enriched biological functions were identified using the candidate genes in each pathway and linkers mediating cross-talk of pathways.
(TIF)

Figure S2 **The survival analysis of two linkers COL4A6 and PLK1.** Two linker genes COL4A6 (p = 0.01) and PLK1 (p = 0.07) were weakly associated with the survival of breast cancer patient.
(TIF)

Figure S3 **An instance of the linker mediating cross-talking pathways.** A linker gene JUN connected leader genes FOS and ETS2 (FOS/JUN/ETS2), mediating cross-talk between “MAPK signaling pathway” and “Dorso-ventral axis formation”. Each ellipse represents a gene in KEGG pathway or ppi network. For a given gene, the ellipse is divided into four parts (from left to right) corresponding to different molecular level with abnormal states (red) in mRNA expression, (green) DNA methylation, (yellow) copy number and blue (somatic mutation), respectively.
(TIF)

Table S1 **A list of differentially expressed genes.**
(XLS)

Table S2 **A list of differentially methylated genes.**
(XLS)

Table S3 **A list of risk pathways.**
(XLS)

Table S4 **A list of cross-talking pathways.**
(XLS)

Table S5 **Linker and leader genes in each cross-talking pathway pair.**
(XLS)
