# Correction: Integrating Multi-Omics for Uncovering the Architecture of Cross-Talking Pathways in Breast Cancer

**DOI:** 10.1371/journal.pone.0221483

**Published:** 2019-08-15

**Authors:** Li Wang, Yun Xiao, Yanyan Ping, Jing Li, Hongying Zhao, Feng Li, Jing Hu, Hongyi Zhang, Yulan Deng, Jiawei Tian, Xia Li

The Materials and Methods section states: "The multi-dimensional breast cancer associated datasets containing 304 human breast cancer samples and 18 non-tumor samples with mRNA expression data, DNA methylation, DNA copy number, and somatic mutation, which were collected from the public database TCGA (available at https://tcga-data.nci.nih.gov/docs/publications/brca_2012/)".

The reported approach is dependent on multi-dimensional data availability. Samples from the TCGA database were selected for inclusion in the study if data was available on all of the following: mRNA expression, DNA methylation, DNA copy number and somatic mutation. For clarification of the dataset used, the authors provide here two new Supporting Information files listing the sample names from the TCGA database that were included in this study. Please note that researchers must apply to the Data Access Committee for the level 2 mutation data.

## Supporting information

S1 File Normal sample names. (TXT)

S2 File BRCA sample names. (TXT)
