# Supplementary figures and images for: Integrating Multi-Omics for Uncovering the Architecture of Cross-Talking Pathways in Breast Cancer
Source: PLoS One. 2014 Aug 19;9(8):e104282. doi: 10.1371/journal.pone.0104282 (PMC4138095; doi:10.1371/journal.pone.0104282)

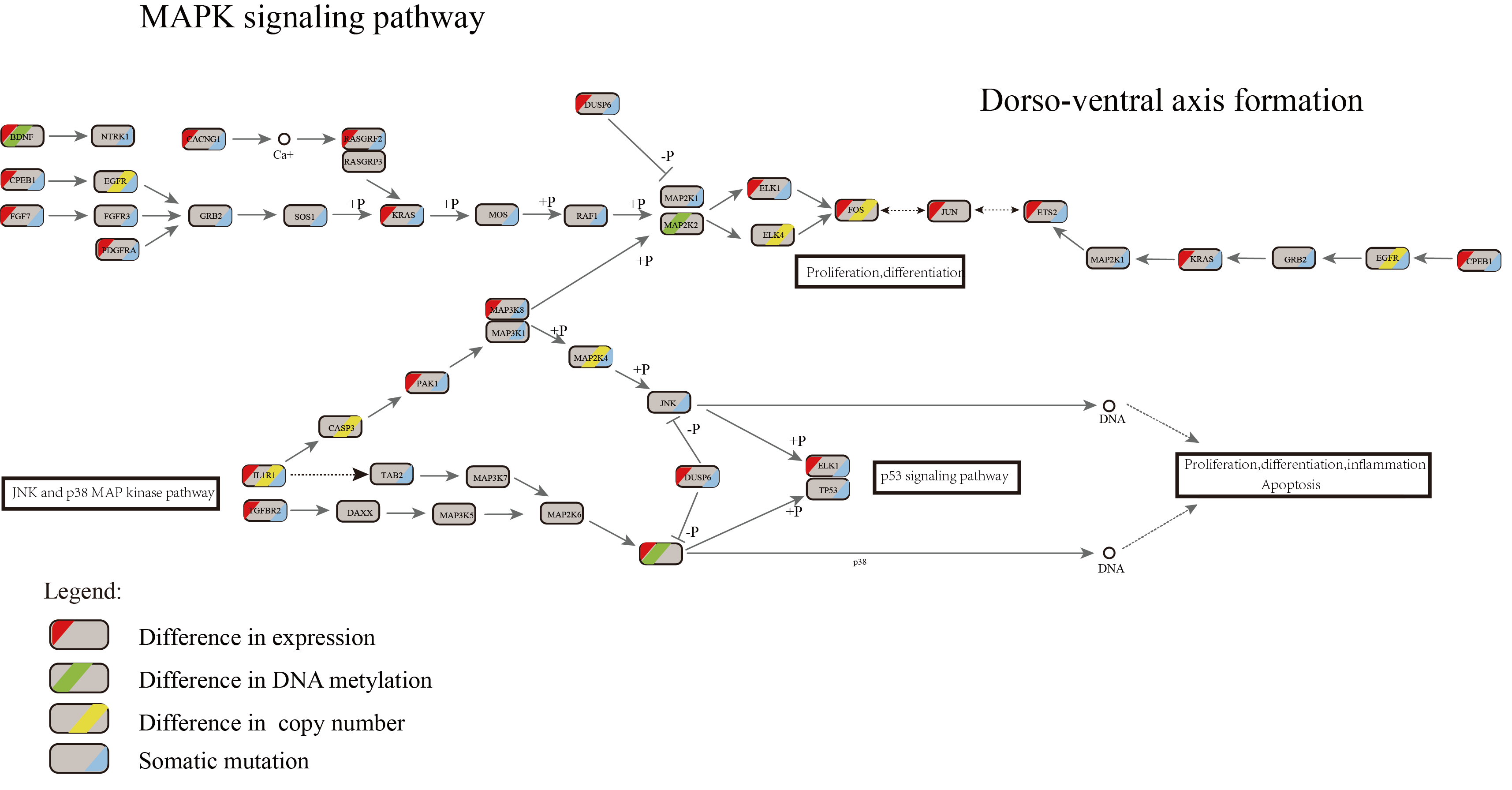

Supplement: Figure S1 — The function network of cross-talking pathways. Based on the structure of cross-talking pathways, the significantly enriched biological functions were identified using the candidate genes in each pathway and linkers mediating cross-talk of pathways. (TIF) [file pone.0104282.s001.tif]

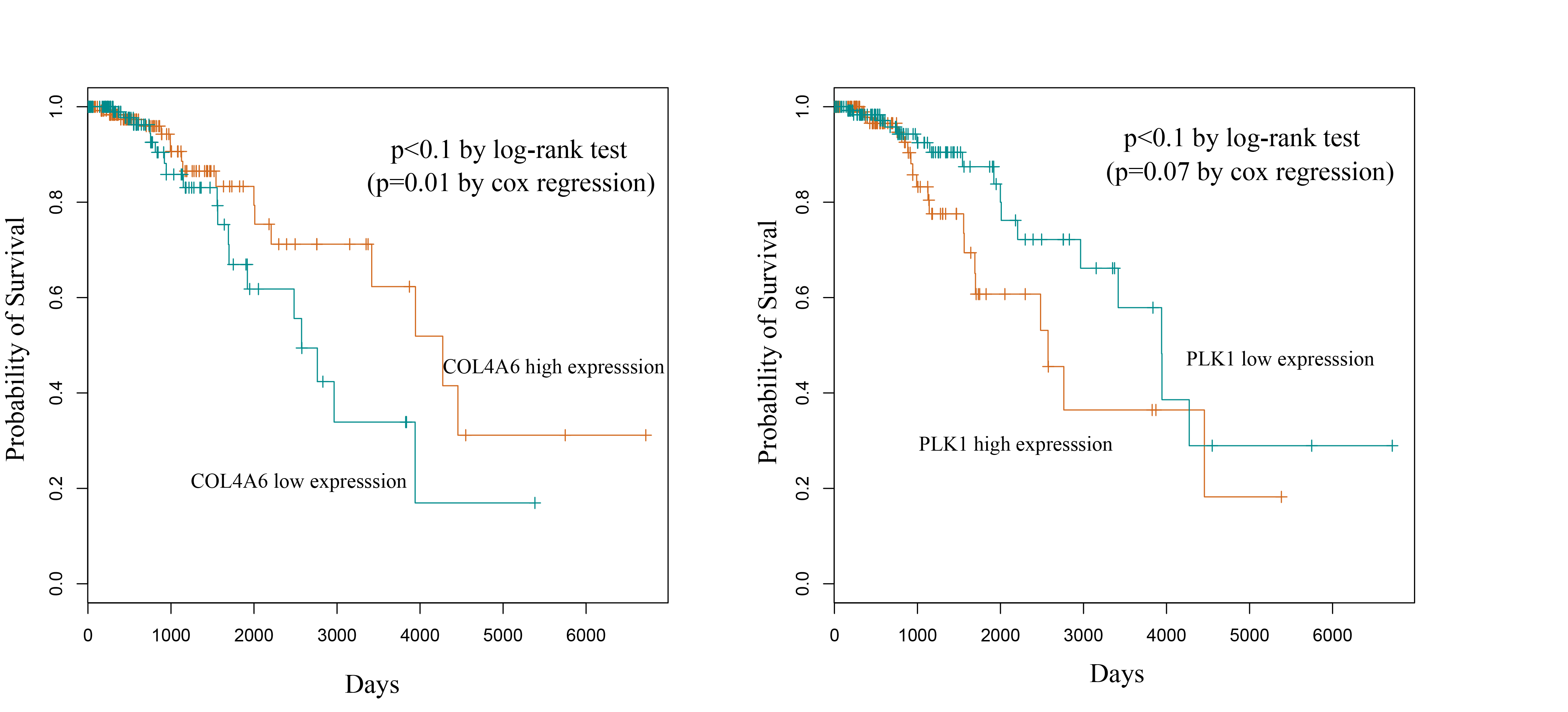

Supplement: Figure S2 — The survival analysis of two linkers COL4A6 and PLK1. Two linker genes COL4A6 (p = 0.01) and PLK1 (p = 0.07) were weakly associated with the survival of breast cancer patient. (TIF) [file pone.0104282.s002.tif]

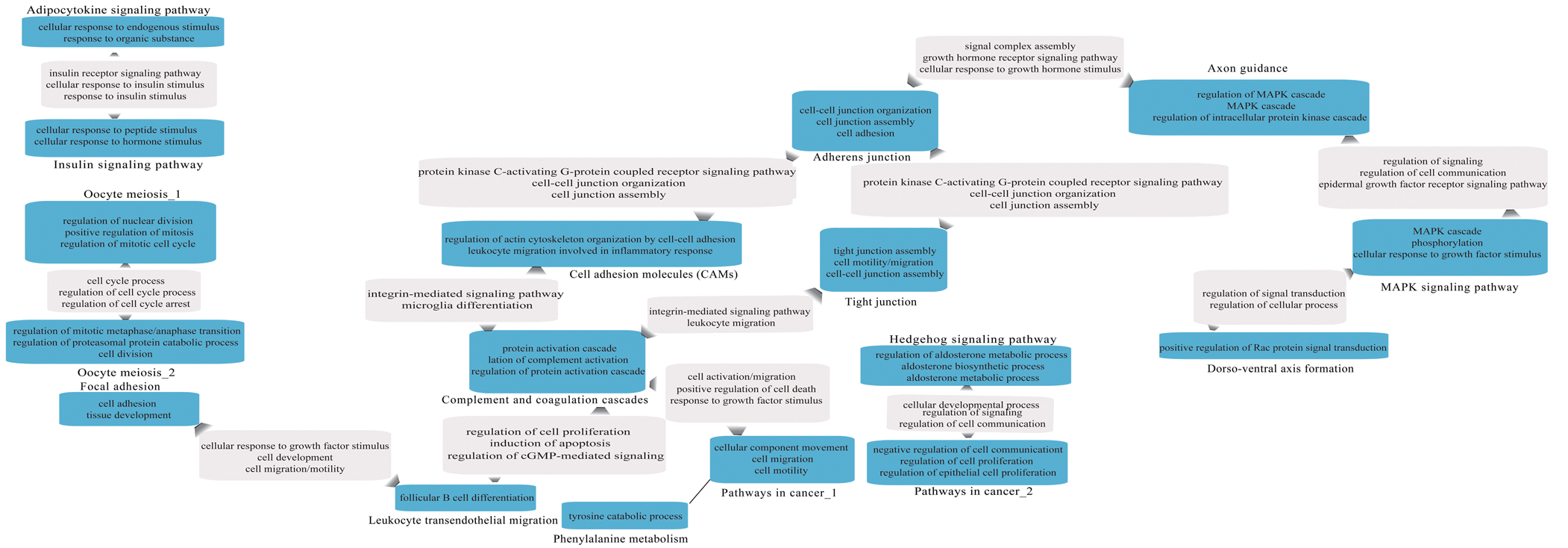

Supplement: Figure S3 — An instance of the linker mediating cross-talking pathways. A linker gene JUN connected leader genes FOS and ETS2 (FOS/JUN/ETS2), mediating cross-talk between “MAPK signaling pathway” and “Dorso-ventral axis formation”. Each ellipse represents a gene in KEGG pathway or ppi network. For a given gene, the ellipse is divided into four parts (from left to right) corresponding to different molecular level with abnormal states (red) in mRNA expression, (green) DNA methylation, (yellow) copy number and blue (somatic mutation), respectively. (TIF) [file pone.0104282.s003.tif]
